# Consistency of hearing aid use, speech perception and vocabulary in hearing impaired children

**DOI:** 10.1590/2317-1782/20242024017en

**Published:** 2024-11-08

**Authors:** Marina Marques dos Santos, Regiane Silva Pereira, Beatriz de Castro Andrade Mendes, Beatriz Calvacanti de Albuquerque Caiuby Novaes

**Affiliations:** 1 Pontifícia Universidade Católica de São Paulo – PUC-SP - São Paulo (SP), Brasil.

**Keywords:** Vocabulary, Speech Perception, Hearing Aids, Hearing Loss, Child

## Abstract

**Purpose:**

To verify the relationship between consistency in the use of hearing aids, auditory speech perception and vocabulary in children using hearing aids.

**Methods:**

The population of Pereira’s (2023) study was resumed and the database was analyzed containing information about the Speech Intelligibility Index (SII) for input sounds of 65 dBSPL (with or without hearing aids) of 29 children with neurosensorial hearing loss and hearing aid users bilaterally, with oral language, the number of hours per day of use of the device, four-tone average, results of the repetition of words with or without meaning in 65 dBSPL, Peabody Picture Vocabulary Test – PPVT. The consistency of the use was analyzed through measuring the auditory dosage which takes into account the average of use hours and the audibility with or without the device.

**Results:**

The PPVT has a positive correlation with the SII with the device at 65 dBSPL. The four-tone average has a significant negative correlation with the SII in both conditions; and the same happens with the hours on daily usage. The dosage has a significant positive correlation with the SII and negative with the PTA.

**Conclusion:**

The receptive vocabulary tends to grow alongside with the audibility dosage increment. Results suggest that listening experience, involving the audibility with or without the device and the consistency of the device daily use must be considered throughout the whole process of the rehabilitation.

## INTRODUCTION

One of the main objectives of early intervention in children with hearing impairment is language acquisition, and the audibility of speech sounds with the use of an Individual Sound Amplification Device (AASI/hearing aid) is a necessary condition for their development since it allows access to linguistic *input*. However, the audibility provided by the hearing aid may be different for each child depending on the degree of hearing loss, adjustments and prescriptive rules used^(1)^. Furthermore, children's auditory experiences vary greatly and are influenced by several factors beyond the audibility of speech sounds, such as cognitive and linguistic abilities, effective use of hearing aids, the linguistic environment in which the child is inserted, and how well their devices are programmed and verified^(2,3)^.

In order to assess the child's performance and progress with their hearing aids, speech-language pathologists use evaluative measures as verification and validation methods. The Speech Intelligibility Index (SII) is one of these objective measures obtained in the hearing aid verification process which ensures access to speech sounds with quality and without discomfort. This measurement can be obtained for speech signals of different intensities – weak, medium and strong – with the dialogic situation occurring at levels considered medium (65 dBSPL), corresponding to a distance of one meter; and the weak speech signals (55 dBSPL) are equivalent to conversations with greater distances between the interlocutors. In addition, it provides the amount of audible speech information that the individual receives with and without the use of sound amplification. Thus, results with low SII indices indicate limited access to speech sounds, which may indicate a risk for delayed vocabulary development^(1-4)^.

Having access to speech sounds in different situations, such as different environments and distances, is essential for children with hearing impairments since much of the learning of new words occurs through incidental listening, that is, when conversations in the environment are not specifically directed at the child. Incidental listening functions as the main gateway to the acquisition of receptive vocabulary and, for oral language to develop is necessary to provide adequate auditory exposure, allowing the child to learn through imitation^(5)^.

For such progress, the child needs to have a considerable amount of time using hearing aids, using them all the time they are awake. On average, the cases with the longest time of use register around 9.2 hours per day^(6)^. The minimum use of hearing aids for 10 hours per day is recommended to promote the development of verbal language since direct and indirect auditory information contributes to the expansion of the linguistic repertoire, taking into account the presence of residual hearing^(7)^.

Although it is crucial to ensure consistent use of hearing aids for proper adaptation, language development and improvement of auditory perception, studies show that this approach represents a delicate variable when trying to relate it to each child's audibility and speech perception^(8-10)^.

A new algorithm, called auditory dosage, was proposed with the aim of quantifying the auditory experience of children using hearing aids, taking into account the audibility of audible speech sounds with and without hearing aids and the frequency with which children use their hearing aids in hours/day since the consistency of use has been calculated^(3,4)^. The hearing dosage considers not only the SII 65 dB with a sound amplification device, which reflects a child's auditory access while using the hearing aid, but also includes access to speech sounds when listening without the hearing aid based on the hours of use of the device, important aspects for audibility in cases of mild and moderate losses.

The concept of “dosage” has been used in previous language intervention research^(11)^ to quantify the intensity and duration of treatment. For children with hearing impairment, “auditory dosage” quantifies individual differences in auditory access, reflecting audibility and the use of hearing aids in a single value with higher auditory dosage values ​​corresponding to more hours of audibility^(3,4)^.

The formula for hearing dosage is calculated by the number of hours the child uses hearing aids per day, raised to the SII with hearing aids, plus the time the child spends without hearing aids, raised to the SII without hearing aids. The time without hearing aids is calculated by considering 24 minus the hours of use per day.


$$\begin{array}{l} Hearing\;dosage\;=\;hours\;of\;use\;per\;day^{\left(SII\;with\;AASI\right)}+time\;without\;hearing\;aids^{\left(SII\;without\;AASI\right)}.\\ \; \end{array}$$ (1)

Time without hearing aids = 24 – hours of use per day.

Although children are not expected to be awake and listening 24 hours a day, this period was used rather than estimating each child's waking hours since sleep time differs^(3)^.

A study^(4)^ indicates that patients who are exposed to a longer period of auditory stimulation demonstrate superior results and improved performance in executive skills, such as working memory and attention, and these skills are directly related to vocabulary development. In addition, children with higher auditory dosage had greater receptive vocabulary than children with lower auditory dosage and it was directly related to measures of verbal working memory.

In the same study, no direct relationship was found between the hours of hearing aid use and the subjects' oral language development, but it was possible to verify that a higher hearing dose is related to better language results. This suggests that the association between hearing dose and language results may be driven more by the variability in SII with and without hearing aids. Furthermore, the relationship between language and hearing dosage is positive up to 10 hours of dosage, but does not increase substantially beyond 10 hours. The nonlinear relationship with language outcomes beyond 10 hours of dosage suggests that additional hours of hearing aid use may not be uniformly beneficial for children, varying from case to case.

In the current research, the population from Pereira’s study^(10)^ was revisited, in which no significant difference was found between the number of hours of daily use of the hearing aid and speech perception and receptive vocabulary skills, probably because it was a group of children with mild to moderate hearing losses and with some audibility for speech sounds without hearing aids. Our objective was to verify whether there is a relationship between consistency of use of hearing aids (auditory dosage), speech perception (SII 65 dB - audibility and discrimination of words with and without meaning) and receptive vocabulary of children using hearing aids, aiming to contribute and strengthen family guidance by promoting greater consistency in the use of these devices and adherence to the therapeutic process.

## METHOD

The research was approved by the Research Ethics Committee of the Postgraduate Studies Program in Human Communication and Health (PEPG) of the Pontifical Catholic University of São Paulo (PUC-SP), through the Brazil Platform (No. 5,441,206). All guardians of the children evaluated were informed about the nature of the research and were instructed to sign the Free and Informed Consent Form (FICF) described in Pereira’s work^(10)^.

The study was carried out at CeAC (Children's Hearing Center), which is part of the Division of Education and Rehabilitation of Communication Disorders (DERDIC) – Specialized Center for Rehabilitation - CER II of the Pontifical Catholic University of São Paulo (PUC-SP).

The subjects of the present research were selected during the audiological monitoring carried out in the service during the year 2022. There were 29 children diagnosed with bilateral sensorineural hearing loss, from mild to severe, with the exception of one subject with mixed loss. All subjects had SII 65 dB greater than or equal to 56%, were users of hearing aids in good working order and verified based on their hearing thresholds, ensuring the precepts of audiological monitoring and conduct of the Care Network for people with hearing loss who require referral. The subjects' ages ranged from 5 to 12 years old, enrolled in regular school with the exception of two subjects in a bilingual school – L1 in Libras – Brazilian Sign Language, using verbal oral language as the main means of communication and who underwent follow-up at the institution during the research collection period; normal otoscopy, normal middle ear function (type A curve), classified in language category stages 4 in which the child builds sentences of 4 or 5 words and begins to use connective elements or in category 5, in which the child builds sentences of more than 5 words using connective elements, conjugating verbs, using plurals, being fluent in oral verbal Portuguese^(12)^.

As described in the study from which the population originated^(10)^, the group is homogeneous in terms of time of use of the devices (most have been hearing aid users for more than one year), socioeconomic level and maternal education. Sixty-nine percent (%) were classified in social classes C1, C2, D or E, and most mothers had completed high school or higher education. Thus, it is considered that the conditions of understanding and guidance after diagnosis are similar.

Children with comorbidities such as syndromes, intellectual disabilities and cognitive delays identified in the medical and speech therapy evaluation were excluded. These alterations could significantly interfere with language development. Children who did not want to carry out the requested activities were not included in the study.

Before the child's consultation, medical records were inspected to select and characterize the subjects who participated in the study: name, current age, time of use of the devices, risk factors and comorbidities, etiology, level of education of the child and parents, socioeconomic status, audiometric thresholds of 500, 1k, 2k and 4k Hz of both ears, determination of the best ear, SII of the best ear with and without hearing aids at 65 dB, measurement of the number of hours/day of hearing aid use. Children with recurrent middle ear alterations were excluded.

On the day of the consultation, an immittance test was performed to ensure that the child had no middle ear alterations, and an audiological evaluation, programming, *datalogging* (a resource available on the research subjects' hearing aids that records the measurement of daily hours of use of the device, and this data can be viewed using the hearing aid brand's *software*) and hearing aid verification were performed. During this verification, SII data was collected at 65 dB, with and without hearing aids.

If the hearing aids presented problems and/or problems with the mold, they were sent for repair and a new appointment was scheduled after the device was returned.

The assessments were applied in the following order: Socioeconomic questionnaire “Brazil Economic Classification Criteria”, from the Brazilian Association of Research Companies (ABEP)^(13)^; Sense word lists; Randomly distributed nonsense word lists – WASP^(14)^. The Peabody Picture Vocabulary Test – PPVT4^(15)^ consists of assessing the receptive vocabulary of adults and children aged 2 years and six months and older. Form A of the PPVT-4 translated into Portuguese was applied to all research subjects. For a quantitative analysis of the results of this test, the standard score was used (ranging from 20 to 160), with the expected average for the age being 100 with a standard deviation of + or - 15. The PPVT-4 was applied last using orofacial reading since it did not aim to evaluate auditory perception for the words presented, but to measure the child's vocabulary repertoire in relation to their peers of the same age, regardless of auditory perception for the word.

The database was analyzed based on the content collected in the different instruments that were described in the procedures of the study by Pereira^(10)^ to be used in the current research. The analysis took into account the following aspects:

✔ Audiological characteristics, being: average of 500, 1k, 2k and 4k Hz (*Pure Tone Average* – PTA); determination of the best ear; average frequency of daily use of the hearing aid in the best ear; SII with and without hearing aid of 65 dB in the best ear; results of speech perception and receptive vocabulary tests;
✔ Calculation of the period without using the hearing aid and the hearing dosage of the best ear at 65 dB for each subject.

To describe the association between the PPVT (response variable) with variables related to audibility and with the percentages of correct answers for the lists of words with and without meaning (explanatory variables), generalized additive models were adjusted. The latter allow modeling unspecified functions of the predictor variables of each, which may be non-linear, producing more accurate predictions of the response variable^(16)^. Smoothing techniques are used to adjust these models, which allow estimating the function that describes the relationship between each of the explanatory variables and the response variable from the data itself. In the present study, the smoothing technique used was the *loess*, and the smoothing parameter (*span*) was chosen based on visual inspection. The relationships between the response variable and the explanatory variables are described visually through graphs, since the relationship between them is not specified in the adjustment process.

A model was adjusted for each of the explanatory variables: meaningful words, meaningful consonants, nonsense words and nonsense consonants at an intensity of 65 dBSPL, SII with and without hearing aids at an intensity of 65 dBSPL, PTA, daily hours of hearing aid use and hearing dosage at 65 dBSPL. An interaction term of age and the explanatory variable considered was added to each model^(4)^. When there was no contribution of the interaction term in explaining the PPVT, the model was adjusted again without its inclusion. The gam function of the R statistical *software* was used to adjust the models.

Furthermore, we tested the hypothesis that the model without interaction is sufficient to explain the data against the alternative hypothesis that the model containing an unspecified function of the interaction term is necessary, for this, we used the F test of the anova function of the R software^(16)^.

From the graphs of the predicted values ​​of the PPVT obtained in the adjustment of the generalized additive models according to the explanatory variables, we identified for each of them, the values ​​corresponding to the PPVT scores 85 and 100, which correspond respectively to -1sd and the expected mean for age.

To quantify the linear relationship between the PPVT and the variables related to audibility and the percentages of correct answers to the lists of words with and without meaning, the Pearson's correlation coefficient was calculated.

## RESULTS

The sample consisted of 29 children using hearing aids, aged 5 to 12 years. Most of the children in the study had an educational level of 4th to 7th grade of elementary school. Those with an educational level of kindergarten to 4th grade of elementary school were aged 5 to 8 years, and those with an educational level of 4th to 7th grade were aged 10 to 12 years.

The median PPVT is equal to 91, that is, at least 50% of the children have a PPVT score greater than or equal to 91. Considering the categorized PPVT, 16 subjects (55.2%) had a score greater than or equal to 85; four (13.8%) had a score greater than or equal to 70 and less than 85 and nine (31.1%) had a score less than 70.

Table 1 shows summary measures for the percentages of correct answers in the lists of words and consonants with and without meaning at an intensity of 65 dBSPL. There are children with a percentage of correct answers of 100% in the words and consonants with meaning.

**Table 1:** Summary measures for the percentage of correct answers in tests of words and consonants with and without meaning at an intensity of 65 dBSPL with hearing aids

Intensity (dBSPL)	Variable	N	Average	Standard deviation	Minimum	Median	Maximum
65	% hits - Words with meaning	28	80.5	18.5	31.8	86.4	100.0
	% hits - Consonants with meaning	29	86.5	16.4	38.6	93.2	100.0
	% hits - Words without meaning	24	59.0	22.8	22.2	61.1	94.4
	% hits - Consonants without meaning	29	69.6	21.5	21.6	75.7	97.3

Table 2 contains summary measurements of the SII with and without hearing aids at 65 dB SPL and the mean hearing thresholds dB HL at 500 Hz, 1000 Hz, 2000 Hz and 4000 Hz (PTA). Summary measurements for the mean number of hours of daily hearing aid use, the daily hours without hearing aids and hearing dosage at 65 dB are found in Table 3.

**Table 2:** Summary measures of the SII measured in conditions with and without hearing aids at an intensity of 65 dBSPL and the average of the hearing thresholds in 500 Hz, 1000 Hz, 2000 Hz e 4000 Hz (PTA)

Variable	N	Average	Standard deviation	Minimum	Median	Maximum
SII with AASI in 65 dB	29	0.79	0.10	0.58	0.80	0.94
SII without AASI in 65 dB	29	0.32	0.25	0.00	0.28	0.89
PTA (dB)	29	50.6	14.2	27	52	80

Caption: AASI = hearing aid; SII = Speech Intelligibility Index; PTA = Pure Tone Average

**Table 3:** Summary measurements of the average number of daily hours of hearing aid use, daily hours without hearing aids and hearing dosage at 65 dB

Variable	N	Average	Standard deviation	Minimum	Median	Maximum
Average daily hours of AASI use	29	7.98	3.78	0.50	8.00	15.20
Daily hours without AASI	29	16.02	3.78	8.80	16.00	23.50
Hearing dosage (65 dB)	29	8.10	3.25	3.02	8.19	18.78

Caption: AASI = hearing aid

Table 4 shows the Pearson correlation coefficients of the PPVT and the percentages of correct answers in the lists of words with and without meaning at 65 dBSPL. The PPVT has a significant positive correlation with all the percentages of correct answers. It can also be observed that, in general, the percentages of correct answers in the different lists are strongly correlated with each other.

**Table 4:** Pearson correlation coefficients of PPVT and variables of the lists of words with and without meaning at 65 dBSPL

		PCS 65	CCS 65	PSS 65	CSS 65
**PPVT**	r	0.40	0.38	0.77	0.67
	p-value	0.033	0.041	<0.001	<0.001
	N	28	29	24	29
**PCS 65 dBSPL**	r	1	0.93	0.67	0.75
	p-value		<0.001	<0,001	<0.001
	N	28	28	24	28
**CCS 65 dBSPL**	r	0.93	1	0.64	0.75
	p-value	<0.001		0.001	<0.001
	N	28	29	24	29
**PSS 65 dBSPL**	r	0.67	0.64	1	0.97
	p-value	<0.001	0.001		<0.001
	N	24	24	24	24
**CSS 65 dBSPL**	r	0.75	0.75	0.97	1
	p-value	<0.001	<0.001	<0.001	
	N	28	29	24	29

Caption: PCS = words with meaning; CCS = consonants with meaning; PSS = words without meaning; CSS = consonants without meaning

The Pearson correlation coefficients of the PPVT and the audibility-related variables presented in Table 5 show that the PPVT has a positive correlation with the SII with hearing aids at 65 dBSPL. There is no significant correlation between the PPVT and SII in the condition without hearing aids. The PPVT has a significant negative correlation with the PTA. A strong positive correlation is also observed between the SII measurements in the 2 experimental conditions. The PTA has a significant negative correlation with the SII in both conditions and the same occurs, in general, with the average daily hours of use. The dosage has a significant positive correlation with the SII and a negative correlation with the PTA.

**Table 5:** Pearson correlation coefficients of PPVT and variables related to audibility

		SII65with	SII65without	PTA	Average Use	Dosage65
**PPVT**	r	0.44	0.24	-0.41	-0.02	0.37
	p-value	0.018	0.206	0.026	0.914	0.048
	N	29	29	29	29	29
**SII65with**	r	1	0.75	-0.95	-0.41	0.71
	p-value		<0.001	<0.001	0.026	<0.001
	N	29	29	29	29	29
**SII65without**	r	0.75	1	-0.84	-0.34	0.78
	p-value	<0.001		<0.001	0.070	<0,001
	N	29	29	29	29	29
**PTA**	r	-0.95	-0.84	1	0.52	-0.69
	p-value	<0.001	<0.001		0.004	<0.001
	N	29	29	29	29	29
**Average use**	r	-0.41	-0.34	0.52	1	0.15
	p-value	0.026	0.070	0.004		0.454
	N	29	29	29	29	29
**Dosage65**	r	0.71	0.78	-0.69	0.15	1
	p-value	<0.001	<0.001	<0.001	0.454	
	N	29	29	29	29	29

Caption: PTA = Pure Tone Average; SII = Speech Intelligibility Index

The results obtained from the adjustment of the generalized linear models are presented below. The relationship of each explanatory variable with the PPVT obtained from the GAM adjustment was represented graphically by a solid blue line. The gray shaded region around the blue line corresponds to the 95% confidence region.

The solid red horizontal line corresponds to the standardized PPVT score cutoff value of 85. The solid red vertical line identifies the value of the explanatory variable associated with the PPVT cutoff value of 85 and the dashed vertical lines correspond to the 95% confidence interval. For the PPVT score cutoff value of 100, the lines are represented in green.

**Figure 1:**
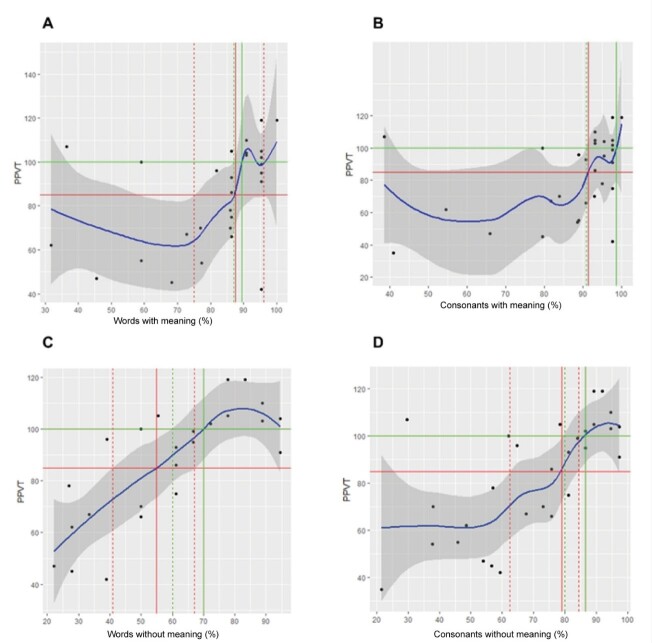
Scatter Plot of PPVT and PCS65

The relationship between the PPVT (receptive vocabulary test) and words with and without meaning (speech perception test) and the PPVT with consonants with and without meaning at 65 dBSPL was represented by means of scatter plots in Figure 1 using a solid blue line.

Caption: Scatter Plot of PPVT and PCS65 with fitted curve and 95% confidence band and PCS65 cutoff values ​​corresponding to PPVT values ​​of 85 and 100 (A) Scatterplot of PPVT and CCS65 with fitted curve and 95% confidence band and CCS65 cutoff values ​​corresponding to PPVT values ​​of 85 and 100 (B) Scatterplot of PPVT and PSS65 with fitted curve and 95% confidence band and PSS65 cutoff values ​​corresponding to PPVT values ​​of 85 and 100 (C) Scatterplot of PPVT and CSS65 with fitted curve and 95% confidence band and CSS65 cutoff values ​​corresponding to PPVT values ​​of 85 and 100 (D). Shaded region = 95% confidence band; red solid horizontal line = PPVT standardized score cutoff value of 85; red solid vertical line = value of the explanatory variable associated with the PPVT cut-off value of 85; dashed vertical lines = confidence interval; green line = PPVT cut-off value of 100

A more pronounced growth trend of the PPVT is observed from a percentage of hits of approximately 75% (Figure 1, graph A). It is noted that in the region where the points are sparse, the confidence band is wider, indicating greater uncertainty in the estimated curve in this region. The percentage of correct answers to meaningful words at 65 dBSPL corresponding to the standardized PPVT score of 85 is 87.5% (95% confidence interval: [75% to 96%]). For the PPVT cutoff value of 100, the cutoff value is 89.5%. In this case, it was only possible to determine the lower confidence limit equal to 87%.

The solid blue line in Figure 1, in the upper right corner (Graph B), represents the relationship between the PPVT and CCS65, the percentage of correct responses for consonants with meaning at 65 dBSPL. A more pronounced growth trend is observed for the PPVT from a percentage of correct responses of approximately 85%. The value of the percentage of correct responses for consonants with meaning at 65 dBSPL corresponding to the standardized PPVT score of 85 is 91.5%. However, it was not possible to establish the uncertainty of this value, assessed by the confidence interval since the continuous red horizontal line, corresponding to the PPVT cut-off value of 85 did not intercept any of the extremes of the confidence band. For the PPVT cut-off value of 100, the cut-off value is 98.5%. In this case, it was only possible to determine the lower confidence limit equal to 91%.

And yet, in Figure 1, in Graph C, the solid blue line represents the relationship between the PPVT and PSS65, meaningless words at 65 dBNPS. A growing trend of PPVT is observed until a percentage of correct answers of approximately 80%. The value of the percentage of correct answers for meaningless words at 65 dBSPL corresponding to the standardized PPVT score of 85 is 55% (95% confidence interval: [41%; 67%]). The cutoff value of PSS65 corresponding to the PPVT value of 100 is 70%. In this case, it was only possible to determine the lower confidence limit equal to 60%.

Figure 1, Graph D, it can be seen that the tendency for the PPVT to increase is more pronounced after a percentage of correct responses of approximately 60%. The value of the percentage of correct responses for meaningless consonants at 65 dBSPL, corresponding to the standardized PPVT score of 85 is 90.5% (95% confidence interval: [82%; 95%]). The cutoff value of CSS65, corresponding to the PPVT value of 100 is 96.5%. In this case, it was only possible to determine the lower confidence limit equal to 91.5%.

**Figure 2:**
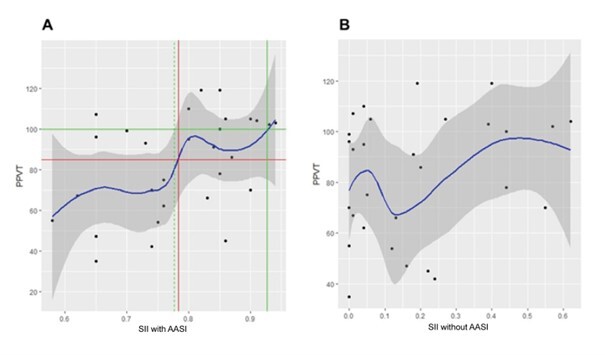
Dispersion diagram of PPVT and SII with hearing aids at 65 dBSPL

Figure 2 shows the relationship between the SII with hearing aids at 65 dBSPL and the SII without hearing aids with PPVT. The adjusted model in Figure 2A indicates a marked increase in PPVT in the range of 0.75 to 0.80 for the SII. The SII value corresponding to a standardized PPVT score of 85 is 0.78 (it was not possible to establish the confidence interval). The SII cutoff value corresponding to a PPVT score of 100 is 0.93. In this case, it was only possible to determine the lower confidence limit equal to 0.78.

Caption: Scatterplot of PPVT and SII with hearing aids at 65 dBSPLwith adjusted curve and 95% confidence band and SII cutoff values ​​corresponding to PPVT values ​​of 85 and 100 (A) Scatterplot of PPVT and SII without hearing aids at 65 dBSPLwith adjusted curve and 95% confidence band (B). Shaded region = 95% confidence band; red solid horizontal line = PPVT standardized score cutoff value of 85; red solid vertical line = value of the explanatory variable associated with the PPVT cutoff value of 85; dashed vertical lines = confidence interval; green line = PPVT score cutoff value of 100

The scatter plot of the PPVT and SII without hearing aids with the fitted curve is shown in Figure 2B. No trends are observed in the point cloud.

**Figure 3:**
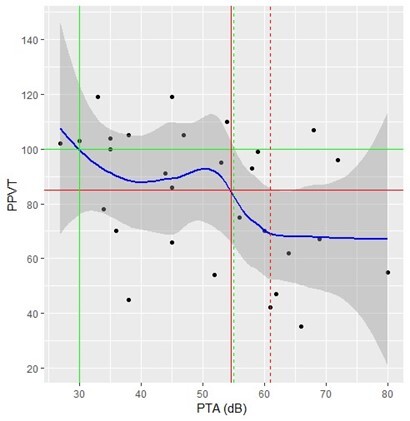
Scatterplot of PPVT and PTA

The fitted curve shown in Figure 3 indicates that the PPVT tends to decrease with increasing the PTA. The PTA value corresponding to a standardized PPVT score of 85 is 54.6 dB (it was only possible to establish the upper limit of the confidence interval which is equal to 61). The PTA cutoff value corresponding to a PPVT value of 100 is 30 dB. In this case, it was only possible to determine the upper confidence limit which was equal to 55 dB.

Caption: Scatter plot of PPVT and PTA with fitted curve and 95% confidence band and PTA cutoff values ​​corresponding to PPVT scores of 85 and 100. Shaded region = 95% confidence band; solid red horizontal line = PPVT standardized score cutoff value of 85; solid red vertical line = value of the explanatory variable associated with the PPVT cutoff value of 85; dashed vertical lines = confidence interval; green line = PPVT score cutoff value of 100

**Figure 4:**
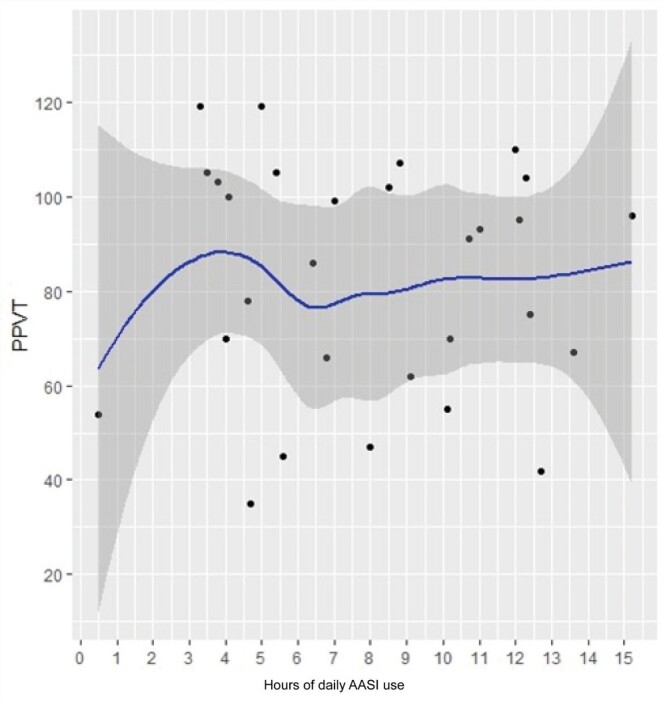
Scatter plot of PPVT and hours of daily hearing aid use

The PPVT scatter plot and daily hours of use with the fitted curve is shown in Figure 4, in which no trends are observed in the point cloud.

Caption: Scatterplot of PPVT and hours of daily hearing aid use with fitted curve and 95% confidence band. Shaded region = 95% confidence band

**Figure 5:**
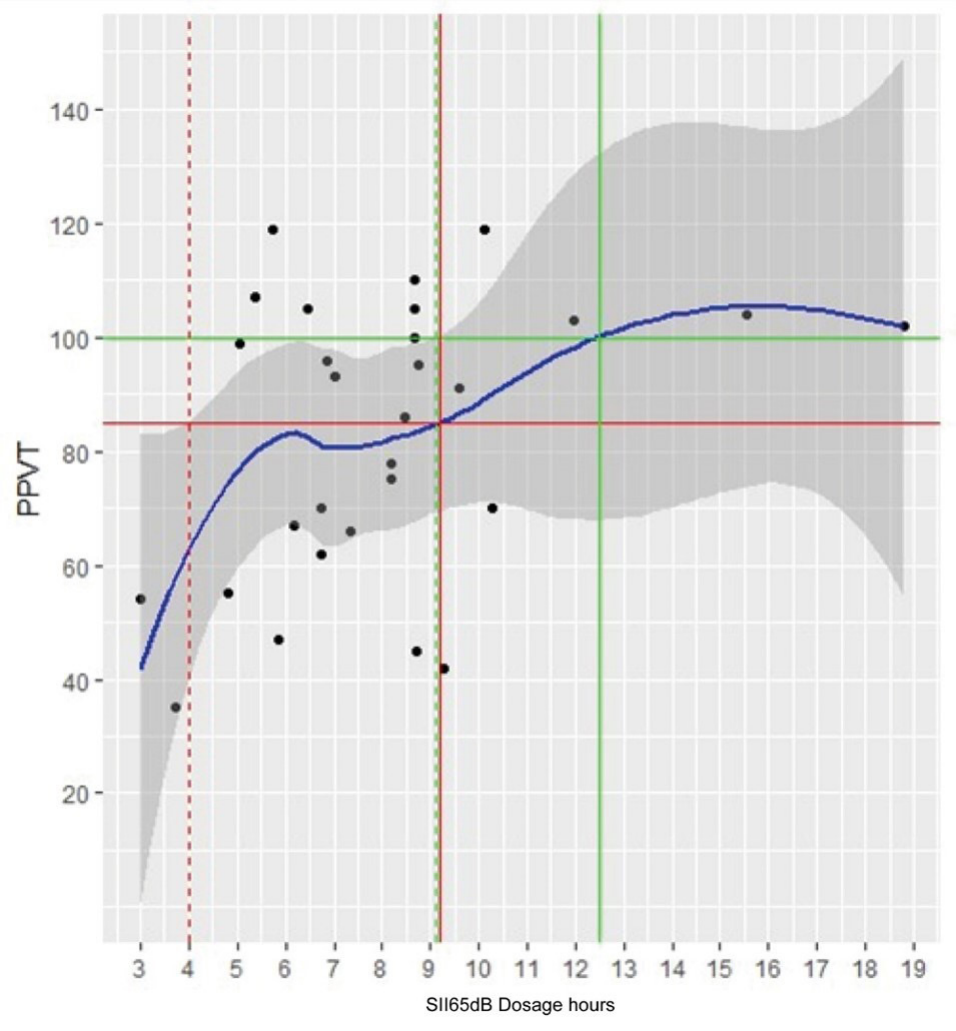
PPVT scatter plot and Dosage65

The fitted curve represented in Figure 5 indicates that the PPVT tends to increase with increasing Dosage 65.

Caption: Scatter Plot of PPVT and Dosage65 with fitted curve and 95% confidence band and dosage cutoff values ​​corresponding to PPVT scores of 85 and 100. Shaded region = 95% confidence band; solid red horizontal line = PPVT standardized score cutoff value of 85; solid red vertical line = explanatory variable value associated with PPVT cutoff value of 85; dashed vertical lines = confidence interval; green line = PPVT score cutoff value of 100

The Dosage 65 value corresponding to the standardized PPVT score of 85 is 9.2 (it was only possible to establish the lower limit of the confidence interval which is equal to 4). The cutoff value of Dosage 65 corresponding to the PPVT value of 100 is 12.5. In this case, it was only possible to determine the lower confidence limit equal to 9.1.

## DISCUSSION

Based on the results and analyzing exclusively the PTA, it is expected that with the increase in the average frequencies there will be lower results in the receptive vocabulary test since these are subjects with greater hearing loss who have less access to speech sounds without the use of devices and consequently lower SII, confirming the need for adherence to the therapeutic process for the development of oral language.

By analyzing the results of the speech perception test, it can be seen that more children were able to discriminate the consonants in the lists with and without meaning than in the lists of words with and without meaning. Furthermore, most were able to respond to the list of words with meaning better than the list of meaningless words. This is due to semantic closure, that is, children are able to perceive the phoneme, but in meaningless words, it is not possible to perform semantic closure to understand the word that was spoken.

In the work that derived from this citation^(10)^, it was described that when lists of meaningless words were presented, the decline in word perception occurred uniformly across the group, evidencing, in this context, the application of the semantic closure strategy in words with meaning, which is not possible with meaningless words when a worsening in the discrimination of consonants is noted.

It is found that as the child's hearing capacity increases, the ability to discriminate will be greater and that the recognition of meaningless words depends almost exclusively on audibility^(17)^.

In the present research, the receptive vocabulary test had a significant positive correlation with the percentages of correct answers in the word lists, so it can be said that as the subjects present better results in the vocabulary test, they will also present better performance in the speech perception test and vice versa.

It is necessary to ensure audibility of speech sounds to provide conditions for auditory skills to develop and, consequently, facilitate the development of oral language. And with the increase in audibility, there is also an increase in receptive vocabulary which probably occurs given the better audibility for speech sounds and better discrimination conditions, determining the SII as a significant predictor in relation to receptive vocabulary^(5)^. As was found in the results of the present research, the receptive vocabulary test had a linear relationship with the SII and the hearing aid. In other words, by ensuring audibility with the use of devices, better results are expected in language tests, as was pointed out in research^(9)^ that subjects with hearing impairment and good audibility are directly related to good vocabulary and reading performance.

From another perspective, the receptive vocabulary test did not have a significant correlation with the SII without hearing aids and no trends are observed in the point cloud in the scatter plot^(18)^. Therefore, the SII without hearing aids may have predictive value but cannot be considered in isolation as an informative diagnosis in identifying children at risk of below-average results^(4)^.

As indicated in other studies^(4,8-10)^, the number of hours of hearing aid use alone is not directly related to language skills. In the present study, it was clear that an index was needed that associated audibility, with and without hearing aids, with the hours of use for the relationship to be significant, that is, the auditory dosage.

To analyze the auditory experience of children considering how much they hear when they are without and with hearing aids and how long this exposure lasts, it is observed that the results of the receptive vocabulary test tend to increase with the increase in the auditory dosage, indicating that the higher the dosage value, the better the receptive language results will be. Similar results were presented in other studies^(3,4)^, whose results are that children with higher auditory dosage present better language results and better receptive vocabulary than children with lower auditory dosage.

It can therefore be suggested that the association between hearing dosage and language outcomes may be driven more by variability in SII with and without hearing aids since the child is also exposed to speech sounds when not using their devices than by an association with the hours of hearing aid use^(4)^.

As demonstrated in the study by the same authors, the relationship between language and auditory dosage is positive in less than 10 hours of dosage, but does not increase substantially above 10 hours of dosage, and children with auditory dosage below 5.3 to 6.7 were at risk of presenting delayed language results. Although multiple instruments were not used to measure language skills, we found similar results here in relation to receptive vocabulary measured by the PPVT.

By ensuring audibility, it is possible to enable/rehabilitate auditory skills and, as a consequence, enable the development of language. Speech therapy is extremely important for children with hearing impairment since it is not enough to just have audibility. Work is also needed so that word recognition can reflect performance compatible with the auditory capacity predicted by the SII, and for this, it is necessary to monitor each child's auditory experience^(17)^.

Since children with better SII without hearing aids can access speech without the device, the usefulness of full-time hearing aid use compared to children who have lower SII without hearing aids is limited, and the consistency of device use is a complex factor that is not directly indicative of effective adherence^(8,10,19)^. Auditory experience or auditory dosage can be used to improve guidance to parents regarding how much the child is hearing in all situations, from the moment they are awake to the moment they are asleep, generating alerts for the need for possible changes in the rehabilitation process.

The collection for the present research was carried out together with another study^(10)^ and was cross-sectional at a certain point in the child's language development, representing one of the limitations in the analysis. Longitudinal studies could measure the effect that hours of hearing aid use have on language development, enabling the identification of individual differences in the use of devices and intervening variables, such as recurrent middle ear changes or broken devices.

The majority of the research subjects (55.2%) presented results within the score in the PPVT test. Among the subjects who presented results below -2 SD in the standardized receptive vocabulary test (31.1%) were S12, who attends a sign language school, and S19 who does not use hearing aids. Among those who presented a result between -1 and -2 dp (13.8%) is S24, a subject with mixed hearing loss who attends a sign language school. Taking into account that the PPVT test represents the daily lives of children, it can be suggested that the subjects who attend the sign language school may not have achieved the test score since the time of exposure to the spoken language is reduced, considering that their daily lives are in sign language.

The fact that both attend a special school in sign language may have interfered with the acquisition of vocabulary in the spoken Portuguese language, considering that both have hearing parents, which means that exposure to sign language occurs mainly in the school environment. The average frequency also does not justify the difference in the test results since S24 has a greater loss than S12, but the type of hearing loss differs. S24 presented better results even though they had mixed hearing loss, that is, they presented a difference between bone conduction and air conduction thresholds, and, in many cases, the conductive component may be responsible for better speech intelligibility.

## CONCLUSION

The present research allowed us to analyze the results of receptive vocabulary and speech perception according to the auditory experience of children with hearing impairment, considering audibility and consistency of use of hearing aids.

The data suggests:

Higher SII 65 dB indices, characteristic of children with audibility for more than 56% of speech sounds, are related to the performance of receptive vocabulary compatible with the age group and speech perception with high performance scores, since by ensuring good audibility for speech sounds, the subjects present better discrimination conditions;
The number of hours of hearing aid use and the SII 65 dB without the devices, in isolation, are not directly related to performance in the receptive vocabulary test. However, when analyzed together, with SII 65 dB with amplification in the calculation of the hearing dosage, they explain the variability in performance in children with minor losses;
The auditory experience, involving audibility with and without hearing aids and the consistency of daily use of the device, must be considered throughout the rehabilitation process, as in addition to indicating and explaining access to sounds in all situations, it also provides guidance elements for parents.

Further research is needed to verify the relationship between auditory dosage and other instruments for analyzing other language skills, in addition to vocabulary, as well as longitudinal studies to measure the effect that hours of use of individual sound amplification devices related to audibility have on the development of oral verbal language.
