# Supplementary material for: Consistency of hearing aid use, speech perception and vocabulary in hearing impaired children
Source: Codas. 2024 Nov 8;36(6):e20240017. doi: 10.1590/2317-1782/20242024017en (PMC11610596; doi:10.1590/2317-1782/20242024017en)
Supplement: Translation

# Translation: Consistência de uso de auxiliares de audição, percepção da fala e vocabulário em crianças com deficiência auditiva

**DOI:** 10.1590/2317-1782/20242024017pt

**Authors:** Marina Marques dos Santos, Regiane Silva Pereira, Beatriz de Castro Andrade Mendes, Beatriz Calvacanti de Albuquerque Caiuby Novaes

**Affiliations:** 1 Pontifícia Universidade Católica de São Paulo – PUC-SP - São Paulo (SP), Brasil.

**Keywords:** Vocabulário, Percepção da Fala, Auxiliares de Audição, Perda Auditiva, Criança

## Abstract

**Objetivo:**

Verificar a relação entre consistência de uso, percepção da fala, e vocabulário receptivo em crianças usuárias de auxiliares de audição, especificamente aparelhos de amplificação sonora individual (AASI).

**Método:**

Foi retomada a população do estudo de Pereira (2023) e analisado o banco de dados com as informações referentes ao Índice de Inteligibilidade de Fala (SII) para sons de entrada de 65 dBNPS (com e sem AASI) de 29 crianças com perda auditiva neurossensorial usuárias de AASI bilateralmente, engajadas em língua oral, em famílias usuárias do português brasileiro. Foram analisados: o número de uso de horas por dia do dispositivo, média quadritonal, resultado da repetição de palavras com e sem sentido em 65 dBNPS, teste Peabody Picture Vocabulary Test – PPVT. A consistência de uso foi analisada por meio da medida da dosagem auditiva, que considera média de horas de uso e audibilidade com e sem AASI.

**Resultados:**

O PPVT tem correlação positiva com o SII com AASI em 65 dBNPS. A média quadritonal tem correlação significativa negativa com o SII nas duas condições e o mesmo acontece com a média de horas diárias de uso. A dosagem tem correlação positiva significativa com os SII e negativa com a PTA.

**Conclusão:**

O vocabulário receptivo tende a crescer com o aumento da dosagem auditiva. Os resultados sugerem que a experiência auditiva, envolvendo a audibilidade sem e com AASI e a consistência de uso diário do dispositivo, deve ser considerada durante todo o processo de reabilitação.

## INTRODUÇÃO

Um dos principais objetivos da intervenção precoce em crianças com deficiência auditiva é a aquisição de linguagem, sendo que a audibilidade dos sons da fala com o uso de Aparelho de Amplificação Sonora Individual (AASI) é uma condição necessária para o seu desenvolvimento, pois permite o acesso ao *input* linguístico. Porém, a audibilidade fornecida pelo AASI pode ser diferente para cada criança dependendo do grau da perda auditiva, ajustes e regras prescritivas utilizadas^(1)^. Além disso, as experiências auditivas das crianças variam muito e são influenciadas por vários fatores além da audibilidade dos sons de fala, como habilidades cognitivas, linguísticas, uso efetivo do AASI, ambiente linguístico em que a criança está inserida e o quão bem seus dispositivos estão programados e verificados^(2,3)^.

Com o objetivo de avaliar o desempenho e progresso da criança com seu AASI, os profissionais de fonoaudiologia se utilizam de medidas avaliativas como métodos de verificação e validação. O Índice de Inteligibilidade de Fala (SII) é uma destas medidas objetivas obtidas no processo de verificação do AASI, que assegura o acesso aos sons de fala com qualidade e sem desconforto. Esta medida pode ser obtida para sinais de fala de diferentes intensidades – fraca, média e forte –, sendo que a situação dialógica ocorre em níveis considerados médios (65 dBNPS), correspondente a uma distância de um metro; e os sinais de fala fraca (55 dBNPS) são equivalentes a conversas de maiores distâncias entre os interlocutores. Além disso, disponibiliza a quantidade de informação de fala audível que o indivíduo recebe com e sem o uso da amplificação sonora, dessa forma, resultados com índices baixos de SII indicam acesso limitado aos sons de fala, podendo indicar risco para atraso no desenvolvimento de vocabulário^(1-4)^.

Ter acesso aos sons da fala em diversas situações, como diferentes ambientes e distâncias, é fundamental para crianças com deficiência auditiva, pois grande parte da aprendizagem de novas palavras ocorre por meio da escuta incidental, ou seja, quando as conversas no ambiente não estão direcionadas especificamente para a criança. A escuta incidental funciona como a principal porta de entrada para a aquisição do vocabulário receptivo e, para que a linguagem oral se desenvolva, é necessário proporcionar uma exposição auditiva adequada, permitindo que a criança possa aprender por meio da imitação^(5)^.

Para tal progresso, a criança precisa ter um tempo considerável de horas de uso dos AASI, utilizando-o durante todo o tempo que está acordada. Em média, os casos com maior tempo de uso registram cerca de 9,2 horas por dia^(6)^. O uso mínimo de 10 horas por dia de AASI é recomendado para favorecer o desenvolvimento da linguagem verbal, pois as informações auditivas diretas e indiretas contribuem para a expansão do repertório linguístico, levando em conta a presença da audição residual^(7)^.

Apesar de ser crucial garantir o uso consistente dos AASI para uma adaptação adequada, desenvolvimento da linguagem e melhoria da percepção auditiva, estudos mostram que essa abordagem representa uma variável delicada ao tentar relacioná-la com a audibilidade e percepção da fala de cada criança^(8-10)^.

Um novo algoritmo, nomeado dosagem auditiva, foi proposto com o objetivo de quantificar a experiência auditiva das crianças usuárias de AASI, levando em consideração a audibilidade dos sons de fala audível com e sem AASI e a frequência com a qual as crianças utilizam seus AASI em horas/dia, conforme tem sido calculada a consistência de uso^(3,4)^. A dosagem auditiva considera não só o SII 65dB com aparelho de amplificação sonora, que reflete o acesso auditivo de uma criança enquanto ela está usando o AASI, mas inclui também o acesso aos sons de fala quando ela está ouvindo sem o AASI, com base nas horas de uso do dispositivo, aspectos importantes para a audibilidade nos casos de perdas leves e moderadas.

O conceito de “dosagem”, foi utilizado em pesquisas anteriores de intervenção de linguagem^(11)^, para quantificar a intensidade e a duração do tratamento. Já para as crianças com deficiência auditiva, a “dosagem auditiva” quantifica as diferenças individuais no acesso auditivo, refletindo a audibilidade e o uso de AASI em um único valor, sendo que maiores os valores de dosagem auditiva, correspondem a mais horas de audibilidade^(3,4)^.

A fórmula da dosagem auditiva é calculada pelo número de horas que a criança usa os AASI por dia, elevado ao SII com AASI, somado ao tempo em que a criança fica sem o AASI, elevado ao SII sem AASI. O tempo sem AASI é calculado considerando 24 menos as horas de uso por dia.


$$Dosagem\;Auditiva\;=\;uso\;de\;horas\;por\;dia^{\left(SII\;com\;AASI\right)}+tempo\;sem\;AASI^{\left(SII\;sem\;AASI\right)}.$$

Tempo sem AASI = 24 – horas de uso por dia.

Embora não haja expectativas que as crianças fiquem acordadas e ouvindo 24 horas por dia, foi utilizado esse período em vez de estimar as horas acordadas de cada criança, pois o tempo de sono difere^(3)^.

Um estudo^(4)^ indica que pacientes que são expostos a um maior tempo de estímulo auditivo demonstram resultados superiores e um desempenho aprimorado nas habilidades executivas, como memória de trabalho e atenção, e essas habilidades estão diretamente relacionadas ao desenvolvimento do vocabulário. Além disso, crianças com maior dosagem auditiva apresentaram maior vocabulário receptivo do que crianças com menor dosagem auditiva e foi diretamente relacionada com medidas de memória de trabalho verbal.

Ainda no mesmo estudo, não se encontrou uma relação direta entre as horas de uso do AASI e o desenvolvimento da linguagem oral dos sujeitos, mas foi possível verificar que maior dosagem auditiva está relacionada a melhores resultados de linguagem. Isto sugere que a associação entre a dosagem auditiva e os resultados de linguagem pode ser impulsionada mais pela variabilidade no SII com e sem o AASI. Além disso, a relação entre linguagem e dosagem auditiva é positiva até 10 horas de dosagem, mas não aumenta substancialmente acima de 10 horas. A relação não linear com resultados de linguagem acima de 10 horas de dosagem sugere que horas adicionais de uso de AASI podem não ser uniformemente benéficas para as crianças, variando em cada caso.

Na atual pesquisa, foi retomada a população do estudo de Pereira^(10)^, na qual não foi encontrada diferença significativa entre número de horas de uso por dia do AASI e as habilidades de percepção de fala e vocabulário receptivo, provavelmente por se tratar de um grupo de crianças com perdas leves e moderadas e com alguma audibilidade para sons de fala sem AASI. Nosso objetivo foi verificar se há relação entre consistência de uso dos AASI (dosagem auditiva), a percepção da fala (SII 65dB - audibilidade e discriminação de palavras com e sem sentido) e vocabulário receptivo de crianças usuárias de AASI, visando a contribuir e fortalecer a orientação familiar promovendo maior consistência no uso desses dispositivos e adesão ao processo terapêutico.

## MÉTODO

A pesquisa foi aprovada pelo Comitê de Ética em Pesquisa do Programa de Estudos Pós-graduados em Comunicação Humana e Saúde (PEPG) da Pontifícia Universidade Católica de São Paulo (PUC-SP), por meio da Plataforma Brasil (No 5.441.206). Todos os responsáveis pelas crianças avaliadas foram informados sobre o caráter da pesquisa, sendo orientados a assinar o Termo de Consentimento Livre e Esclarecido (TCLE) descrito no trabalho de Pereira^(10)^.

O estudo foi realizado no CeAC (Centro Audição na Criança), que faz parte da Divisão de Educação e Reabilitação dos Distúrbios da Comunicação (DERDIC) – Centro Especializado em Reabilitação - CER II da Pontifícia Universidade Católica de São Paulo (PUC-SP).

Os sujeitos dessa pesquisa foram selecionados durante o acompanhamento audiológico realizado no serviço no decorrer do ano de 2022. Foram 29 crianças com diagnóstico de deficiência auditiva neurossensorial bilateral, de grau leve a severo, com exceção de um sujeito de perda mista. Todos os sujeitos tinham SII 65 dB maior ou igual a 56%, eram usuários de AASI em bom funcionamento e verificados a partir de seus limiares auditivos, assegurados os preceitos de acompanhamento audiológico e condutas da Rede de Cuidados à pessoa com deficiência auditiva que necessita da referência. A faixa etária dos sujeitos variou entre 5 e 12 anos, matriculados na escola regular com exceção de dois sujeitos em escola bilíngue – L1 em Libras – Língua Brasileira de Sinais, utilizando a língua oral verbal como principal meio de comunicação e que realizaram acompanhamento na instituição no período de coleta da pesquisa; otoscopia normal, função normal da orelha média (curva tipo A), classificados nos estágios de categorias de linguagem 4, na qual a criança constrói frases de 4 ou 5 palavras, e inicia o uso de elementos conectivos ou na categoria 5 em que a criança constrói frases de mais de 5 palavras usando elementos conectivos, conjugando verbos, usando plurais, sendo fluente na língua portuguesa oral verbal^(12)^.

Como descrito no trabalho do qual a população se origina^(10)^, o grupo é homogêneo quanto ao tempo de uso dos dispositivos (maioria são usuários de AASI há mais de um ano), ao nível socioeconômico e escolaridade materna. Sessenta e nove porcento (%) foram classificados nas classes sociais C1, C2, D ou E e a maioria das mães cursou o ensino médio ou superior. Assim, considera-se que as condições de compreensão e orientação após o diagnóstico são similares.

Foram excluídas crianças com comorbidades como síndromes, deficiência intelectual e atrasos cognitivos, identificados na avaliação médica e fonoaudiológica. Essas alterações poderiam interferir significativamente no desenvolvimento de linguagem. Crianças que não quiseram realizar as atividades solicitadas não foram incluídas no estudo.

Antes da consulta da criança foi realizada a inspeção de prontuários para seleção e caracterização dos sujeitos que participaram da pesquisa: nome, idade atual, tempo de uso dos dispositivos, fatores de risco e comorbidades, etiologia, nível de escolaridade da criança e dos pais, nível socioeconômicos, limiares audiométricos de 500, 1k, 2k e 4k Hz de ambas as orelhas, determinação da melhor orelha, SII da melhor orelha com e sem AASI em 65 dB, medida do número de horas/dia de uso dos AASI. Foram excluídas crianças com alterações de orelha média recorrentes.

No dia da consulta foi realizada a imitanciometria para garantir que a criança estava sem alterações de orelha média e realizada a avaliação audiológica, programação, coleta do *datalogging (*recurso disponível nos AASI dos sujeitos da pesquisa que registra a medida de uso de horas diárias do dispositivo, sendo possível visualizar esse dado por meio do *software* da marca do AASI) e verificação dos AASI. Durante esta verificação foi realizada a coleta dos dados de SII em 65 dB, com e sem AASI.

Caso os AASI apresentassem problemas e/ou problemas com o molde, eram enviados ao conserto e agendada nova consulta após o retorno do dispositivo.

As avaliações foram aplicadas na seguinte ordem: Questionário socioeconômico “Critério de Classificação Econômica Brasil”, da Associação Brasileira de Empresas de Pesquisa (ABEP)^(13)^; Listas de palavras com sentido; Listas de palavras sem sentido previamente distribuídas aleatoriamente – WASP^(14)^. O Peabody Picture Vocabulary Test – PPVT4^(15)^ consiste na avaliação do vocabulário receptivo de adultos e crianças a partir de 2 anos e seis meses. O Formulário A do PPVT-4 traduzido para a língua portuguesa foi aplicado em todos os sujeitos da pesquisa. Para uma análise quantitativa dos resultados deste teste, foi utilizado o escore padrão (que vai de 20 a 160), sendo a média esperada para a idade 100, com desvio padrão de + ou – 15. O PPVT-4 foi aplicado por último, com utilização de leitura orofacial, pois não teve como objetivo avaliar a percepção auditiva para as palavras apresentadas, mas aferir o repertório de vocábulos da criança em relação a seus pares da mesma idade, independentemente da percepção auditiva para a palavra.

Foi analisado o banco de dados a partir do conteúdo coletado nos diferentes instrumentos que foram descritos nos procedimentos do estudo de Pereira^(10)^ para serem utilizados na atual pesquisa. A análise levou em consideração os seguintes aspectos:

✔ Características audiológicas, sendo: média quadritonal de 500, 1k, 2k e 4k Hz (*Pure Tone Average* – PTA); determinação da melhor orelha; média da frequência do uso diário do AASI na melhor orelha; SII com e sem AASI de 65 dB da melhor orelha; Resultados de testes de percepção da fala e de vocabulário receptivo;
✔ Cálculo do período sem uso do AASI e da dosagem auditiva da melhor orelha em 65 dB de cada sujeito.

Para descrever a associação entre o PPVT (variável resposta) com variáveis relacionadas à audibilidade e com as porcentagens de acertos das listas de palavras com e sem sentido (variáveis explicativas) foram ajustados modelos aditivos generalizados. Estes últimos permitem modelar funções não especificadas das variáveis preditoras de cada, que podem ser não lineares, produzindo previsões mais precisas da variável resposta^(16)^. No ajuste desses modelos são utilizadas técnicas de suavização, que permitem estimar a função que descreve a relação entre cada uma das variáveis explicativas e a variável resposta a partir dos próprios dados. Neste estudo, a técnica de suavização utilizada foi a *loess*, e o parâmetro de suavização (*span*) foi escolhido a partir de inspeção visual. As relações entre a variável resposta e as explicativas são descritas visualmente por meio de gráficos, uma vez que a relação entre elas não é especificada no processo de ajuste.

Foi ajustado um modelo para cada uma das variáveis explicativas: palavras com sentido, consoantes com sentido, palavras sem sentido e consoantes sem sentido na intensidade de 65 dBNPS, SII com e sem AASI na intensidade de 65 dBNPS, PTA, horas diárias de uso de AASI e dosagem auditiva em 65 dBNPS. A cada modelo foi adicionado um termo de interação da idade e a variável explicativa considerada^(4)^. Quando não houve contribuição do termo de interação na explicação do PPVT, o modelo foi ajustado novamente sem a sua inclusão. No ajuste dos modelos foi utilizada a função *gam* do *software* estatístico R.

Além disso, testamos a hipótese de que o modelo sem interação é suficiente para explicar os dados contra a hipótese alternativa de que o modelo que contém uma função não especificada do termo de interação é necessário, para isto, utilizamos o teste F da função anova do software R^(16)^.

A partir dos gráficos dos valores previstos do PPVT obtidos no ajuste dos modelos aditivos generalizados em função das variáveis explicativas identificamos, para cada uma delas, os valores correspondentes aos escores 85 e 100 do PPVT, que correspondem respectivamente a -1dp e média esperada para a idade.

Para quantificar a relação linear entre o PPVT e as variáveis relacionadas à audibilidade e com as porcentagens de acertos das listas de palavras com e sem sentido foi calculado o coeficiente de correlação de Pearson.

## RESULTADOS

A amostra consiste em 29 crianças usuárias de AASI, com idade de 5 a 12 anos. A maioria das crianças da pesquisa tem nível educacional do 4º ao 7º ano do Ensino Fundamental. Naqueles com nível educacional da Educação infantil ao 4º ano do Ensino Fundamental a idade variou de 5 a 8 anos e nos com nível educacional do 4º ao 7º ano, a idade variou de 10 a 12 anos.

A mediana do PPVT é igual a 91, ou seja, pelo menos 50% das crianças têm escore do PPVT maior ou igual a 91. Considerando o PPVT categorizado, 16 sujeitos (55,2%) tiveram escore maior ou igual a 85; quatro (13,8%) tiveram escore maior ou igual a 70 e menor que 85 e nove (31,1%) tiveram escore inferior a 70.

Na Tabela 1 são encontradas medidas resumo para as porcentagens de acertos nas listas de palavras e consoantes com e sem sentido na intensidade de 65 dBNPS. Há crianças com porcentagem de acertos de 100% nas palavras e consoantes com sentido.

**Tabela 1:** Medidas resumo para a porcentagem de acertos nos testes de palavras e consoantes com e sem sentido na intensidade de 65 dBNPS, com AASI

Intensidade (dBNPS)	Variável	N	Média	Desvio padrão	Mínimo	Mediana	Máximo
65	% acertos - Palavras com sentido	28	80,5	18,5	31,8	86,4	100
	% acertos -Consoantes com sentido	29	86,5	16,4	38,6	93,2	100
	% acertos -Palavras sem sentido	24	59	22,8	22,2	61,1	94,4
	% acertos -Consoantes sem sentido	29	69,6	21,5	21,6	75,7	97,3

A Tabela 2 contém medidas resumo do SII, com e sem AASI, em 65 dBNPS e da média dos limiares auditivos dBNA em 500 Hz, 1000 Hz, 2000 Hz e 4000 Hz (PTA). Medidas resumo para a média do número de horas diárias de uso do AASI, das horas diárias sem AASI e dosagem auditiva em 65 dB são encontradas na Tabela 3.

**Tabela 2:** Medidas resumo do SII medido nas condições com e sem AASI na intensidade de 65 dBNPS e da média dos limiares auditivos em 500 Hz, 1000 Hz, 2000 Hz e 4000 Hz (PTA)

Variável	N	Média	Desvio padrão	Mínimo	Mediana	Máximo
SII com AASI em 65 dB	29	0,79	0,1	0,58	0,8	0,94
SII sem AASI em 65 dB	29	0,32	0,25	0	0,28	0,89
PTA (dB)	29	50,6	14,2	27	52	80

**Tabela 3:** Medidas resumo da média do número de horas diárias de uso do AASI, das horas diárias sem AASI e dosagem auditiva em 65 dB

Variável	N	Média	Desvio padrão	Mínimo	Mediana	Máximo
Média horas diárias de uso do AASI	29	7,98	3,78	0,5	8	15,2
Horas diárias sem AASI	29	16,02	3,78	8,8	16	23,5
Dosagem auditiva (65 dB)	29	8,1	3,25	3,02	8,19	18,78

Na Tabela 4 são encontrados os coeficientes de correlação de Pearson do PPVT e as porcentagens de acertos nas listas de palavras com e sem sentido em 65 dBNPS. O PPVT tem correlação positiva significativa com todas as porcentagens de acertos. Observa-se ainda que, de uma forma geral, as porcentagens de acertos nas diferentes listas são fortemente correlacionadas entre si.

**Tabela 4:** Coeficientes de correlação de Pearson do PPVT e as variáveis das listas de palavras com e sem sentido em 65 dBNPS

		PCS 65	CCS 65	PSS 65	CSS 65
**PPVT**	r	0,4	0,38	0,77	0,67
	valor-p	0,033	0,041	<0,001	<0,001
	N	28	29	24	29
**PCS 65 dBNPS**	r	1	0,93	0,67	0,75
	valor-p		<0,001	<0,001	<0,001
	N	28	28	24	28
**CCS 65 dBNPS**	r	0,93	1	0,64	0,75
	valor-p	<0,001		0,001	<0,001
	N	28	29	24	29
**PSS 65 dBNPS**	r	0,67	0,64	1	0,97
	valor-p	<0,001	0,001		<0,001
	N	24	24	24	24
**CSS 65 dBNPS**	r	0,75	0,75	0,97	1
	valor-p	<0,001	<0,001	<0,001	
	N	28	29	24	29

**Legenda:** PCS = palavras com sentido; CCS = consoantes com sentido; PSS = palavras sem sentido; CSS = consoantes sem sentido

Os coeficientes de correlação de Pearson do PPVT e as variáveis relacionadas à audibilidade apresentados na Tabela 5 mostram que o PPVT tem correlação positiva com o SII com AASI em 65 dBNPS. Não há correlação significativa do PPVT e o SII na condição sem AASI. O PPVT tem correlação significativa negativa com a PTA. Observa-se ainda forte correlação positiva entre as medidas do SII nas 2 condições experimentais. A PTA tem correlação significativa negativa com o SII nas 2 condições e o mesmo acontece, de uma forma geral, com a média de horas diárias de uso. A dosagem tem correlação positiva significativa com os SII e negativa com a PTA.

**Tabela 5:** Coeficientes de correlação de Pearson do PPVT e as variáveis relacionadas à audibilidade

		SII65com	SII65sem	PTA	Média Uso	Dosagem65
**PPVT**	r	0,44	0,24	-0,41	-0,02	0,37
	valor-p	0,018	0,206	0,026	0,914	0,048
	N	29	29	29	29	29
**SII65com**	r	1	0,75	-0,95	-0,41	0,71
	valor-p		<0,001	<0,001	0,026	<0,001
	N	29	29	29	29	29
**SII65sem**	r	0,75	1	-0,84	-0,34	0,78
	valor-p	<0,001		<0,001	0,07	<0,001
	N	29	29	29	29	29
**PTA**	r	-0,95	-0,84	1	0,52	-0,69
	valor-p	<0,001	<0,001		0,004	<0,001
	N	29	29	29	29	29
**Média Uso**	r	-0,41	-0,34	0,52	1	0,15
	valor-p	0,026	0,07	0,004		0,454
	N	29	29	29	29	29
**Dosagem65**	r	0,71	0,78	-0,69	0,15	1
	valor-p	<0,001	<0,001	<0,001	0,454	
	N	29	29	29	29	29

A seguir são apresentados os resultados obtidos no ajuste dos modelos lineares generalizados. A relação de cada variável explicativa com o PPVT obtida a partir do ajuste do GAM foi representada graficamente por uma linha contínua azul. A região sombreada em cinza em torno da linha azul corresponde à região de confiança de 95%.

A linha horizontal contínua vermelha corresponde ao valor de corte do escore padronizado do PPVT de 85. A linha vertical contínua vermelha identifica o valor da variável explicativa associado ao valor de corte do PPVT de 85 e as linhas verticais tracejadas correspondem ao intervalo de confiança de 95%. Para o valor de corte de 100 do escore do PPVT as linhas estão representadas em verde.

Foi representado por meio de gráficos de dispersão a relação do PPVT (teste de vocabulário receptivo), com palavras com e sem sentido (teste de percepção de fala) e do PPVT com consoantes com e sem sentido em 65 dBNPS na Figura 1, por meio de linha azul contínua.

**Figura 1:** Diagrama de dispersão do PPVT e PCS65

Legenda: Diagrama de dispersão do PPVT e PCS65 com curva ajustada e banda de confiança de 95% e valores de corte da PCS65 correspondentes aos valores de 85 e 100 do PPVT (A) Diagrama de dispersão do PPVT e CCS65 com curva ajustada e banda de confiança de 95% e valores de corte da CCS65 correspondentes aos valores de 85 e 100 do PPVT (B) Diagrama de dispersão do PPVT e PSS65 com curva ajustada e banda de confiança de 95% e valores de corte da PSS65 correspondentes aos valores de 85 e 100 do PPVT (C) Diagrama de dispersão do PPVT e CSS65 com curva ajustada e banda de confiança de 95% e valores de corte da CSS65 correspondentes aos valores de 85 e 100 do PPVT (D). Região sombreada = banda de confiança de 95%; linha horizontal contínua vermelha = valor de corte do escore padronizado do PPVT de 85; linha vertical contínua vermelha = valor da variável explicativa associado ao valor de corte do PPVT de 85; linhas verticais tracejadas = intervalo de confiança; linha verde = valor de corte de 100 do escore do PPVT

Observa-se uma tendência de crescimento mais acentuada do PPVT a partir de uma porcentagem de acertos de aproximadamente 75% (Figura 1, gráfico A). Nota-se que na região em os pontos são esparsos, a banda de confiança é mais larga, indicando maior incerteza na curva estimada nessa região. O valor da porcentagem de acertos nas palavras com sentido em 65 dBNPS correspondente ao escore padronizado do PPVT de 85 é 87,5% (intervalo de confiança de 95%: [75% a 96%]). Para o valor de corte de 100 no PPVT, o valor de corte é 89,5%. Neste caso, só foi possível determinar o limite inferior de confiança igual a 87%.

A linha azul contínua na Figura 1, no canto superior direito (gráfico B) representa a relação entre PPVT com CCS65, porcentagem de acerto de consoantes com sentido a 65 dBNPS. Observa-se uma tendência de crescimento mais acentuada do PPVT a partir de uma porcentagem de acertos de aproximadamente 85%. O valor da porcentagem de acertos nas consoantes com sentido em 65 dBNPS correspondente ao escore padronizado do PPVT de 85 é 91,5%. Entretanto, não foi possível estabelecer a incerteza desse valor, avaliada pelo intervalo de confiança, uma vez que a linha horizontal contínua vermelha, correspondente ao valor de corte do PPVT de 85 não interceptou nenhum dos extremos da banda de confiança. Para o valor de corte de 100 no PPVT, o valor de corte é 98,5%. Neste caso, só foi possível determinar o limite inferior de confiança igual a 91%.

Ainda na Figura 1, no gráfico C, a linha azul contínua representa a relação entre PPVT com PSS65, palavras sem sentido a 65 dBNPS. Observa-se uma tendência de crescimento do PPVT até de uma porcentagem de acertos de aproximadamente 80%. O valor da porcentagem de acertos nas palavras sem sentido em 65 dBNPS correspondente ao escore padronizado do PPVT de 85 é 55% (intervalo de confiança de 95%: [41%; 67%]). O valor de corte da PSS65 correspondente ao valor de 100 do PPVT é 70%. Neste caso, só foi possível determinar o limite inferior de confiança igual a 60%.

Observa-se, na Figura 1, gráfico D, que tendência de aumento do PPVT é mais acentuada a partir de uma porcentagem de acertos de aproximadamente 60%. O valor da porcentagem de acertos nas consoantes sem sentido em 65 dBNPS correspondente ao escore padronizado do PPVT de 85 é 90,5% (intervalo de confiança de 95%: [82%; 95%]). O valor de corte da CSS65 correspondente ao valor de 100 do PPVT, é 96,5%. Neste caso, só foi possível determinar o limite inferior de confiança igual a 91,5%.

Na Figura 2 é apresentado a relação do SII com AASI em 65 dBNPS e do SII sem AASI com o PPVT. O modelo ajustado na Figura 2A indica um aumento acentuado do PPVT no intervalo de 0,75 a 0,80 do SII. O valor do SII correspondente ao escore padronizado do PPVT de 85 é 0,78 (não foi possível estabelecer o intervalo de confiança). O valor de corte do SII correspondente ao valor de 100 do PPVT é 0,93. Neste caso, só foi possível determinar o limite inferior de confiança igual a 0,78.

**Figura 2:** Diagrama de dispersão do PPVT e o SII com AASI em 65 dBNPS

Legenda: Diagrama de dispersão do PPVT e o SII com AASI em 65 dBNPS com curva ajustada e banda de confiança de 95% e valores de corte do SII correspondentes aos valores de 85 e 100 do PPVT (A) Diagrama de dispersão do PPVT e o SII sem AASI em 65 dBNPS com curva ajustada e banda de confiança de 95% (B). Região sombreada = banda de confiança de 95%; linha horizontal contínua vermelha = valor de corte do escore padronizado do PPVT de 85; linha vertical contínua vermelha = valor da variável explicativa associado ao valor de corte do PPVT de 85; linhas verticais tracejadas = intervalo de confiança; linha verde = valor de corte de 100 do escore do PPVT

O diagrama de dispersão do PPVT e o SII sem AASI com a curva ajustada é apresentado na Figura 2B. Não são observadas tendências na nuvem de pontos.

A curva ajustada representada na Figura 3 indica que o PPVT tende a diminuir com o aumento da PTA. O valor da PTA correspondente ao escore padronizado do PPVT de 85 é 54,6 dB (só foi possível estabelecer o limite superior do intervalo de confiança que é igual a 61). O valor de corte da PTA correspondente ao valor de 100 do PPVT, é 30 dB. Neste caso, só foi possível determinar o limite superior de confiança igual a 55 dB.

**Figura 3:** Diagrama de dispersão do PPVT e a PTA

Legenda: Diagrama de dispersão do PPVT e a PTA com curva ajustada e banda de confiança de 95% e valores de corte da PTA correspondentes aos valores de 85 e 100 do PPVT. Região sombreada = banda de confiança de 95%; linha horizontal contínua vermelha = valor de corte do escore padronizado do PPVT de 85; linha vertical contínua vermelha = valor da variável explicativa associado ao valor de corte do PPVT de 85; linhas verticais tracejadas = intervalo de confiança; linha verde = valor de corte de 100 do escore do PPVT

O diagrama de dispersão do PPVT e as horas diárias de uso com a curva ajustada é apresentado na Figura 4, no qual não são observadas tendências na nuvem de pontos.

**Figura 4:** Diagrama de dispersão do PPVT e as horas de uso diário do AASI

Legenda: Diagrama de dispersão do PPVT e as horas de uso diário do AASI com curva ajustada e banda de confiança de 95%. Região sombreada = banda de confiança de 95%

A curva ajustada representada na Figura 5 indica que o PPVT tende a aumentar com o aumento da Dosagem65.

**Figura 5:** Diagrama de dispersão do PPVT e a Dosagem65

Legenda: Diagrama de dispersão do PPVT e a Dosagem65 com curva ajustada e banda de confiança de 95% e valores de corte da dosagem correspondentes aos valores de 85 e 100 do PPVT. Região sombreada = banda de confiança de 95%; linha horizontal contínua vermelha = valor de corte do escore padronizado do PPVT de 85; linha vertical contínua vermelha = valor da variável explicativa associado ao valor de corte do PPVT de 85; linhas verticais tracejadas = intervalo de confiança; linha verde = valor de corte de 100 do escore do PPVT

O valor da Dosagem65 correspondente ao escore padronizado do PPVT de 85 é 9,2 (só foi possível estabelecer o limite inferior do intervalo de confiança que é igual a 4). O valor de corte da Dosagem65 correspondente ao valor de 100 do PPVT é 12,5. Neste caso, só foi possível determinar o limite inferior de confiança igual a 9,1.

## DISCUSSÃO

A partir dos resultados e analisando exclusivamente a PTA, espera-se que com o aumento da média das frequências há resultados mais baixos no teste de vocabulário receptivo, pois são sujeitos com perdas auditivas maiores que possuem menos acesso aos sons de fala sem o uso dos dispositivos e consequentemente SII mais baixos, confirmando a necessidade de adesão ao processo terapêutico para desenvolvimento da língua oral.

Com a análise dos resultados do teste de percepção de fala, observa-se que há mais crianças que conseguiram discriminar as consoantes das listas com e sem sentido do que das palavras com e sem sentido. Além disso, a maioria conseguiu responder a lista de palavras com sentido melhor do que a lista de palavras sem sentido. Isso se deve ao fechamento semântico, ou seja, as crianças conseguem ter a percepção do fonema, mas, nas palavras sem sentido, não é possível fazer o fechamento semântico para compreender a palavra que foi dita.

No trabalho que este derivou^(10)^ foi descrito que quando as listas de palavras sem sentido foram apresentadas, o declínio na percepção da palavra ocorre de maneira uniforme em todo o grupo, evidenciando, nesse contexto, a aplicação da estratégia de fechamento semântico – nas palavras com sentido, o que não é possível nas palavras sem sentido –, quando se nota a piora na discriminação de consoantes – das palavras sem sentido.

Constata-se que, conforme a capacidade auditiva da criança aumenta, maior será a habilidade para discriminar e que o reconhecimento de palavras sem sentido depende, quase exclusivamente, da audibilidade^(17)^.

Nesta pesquisa, o teste de vocabulário receptivo teve correlação positiva significativa com as porcentagens de acertos nas listas de palavras, assim pode-se dizer que conforme o sujeito apresenta resultados melhores no teste de vocabulário, apresentará também melhor desempenho no teste de percepção de fala e vice-versa.

É necessário garantir a audibilidade aos sons de fala para fornecer condições para que as habilidades auditivas se desenvolvam e, por consequência, facilitar o desenvolvimento da língua oral. E com o aumento da audibilidade também há aumento do vocabulário receptivo, o que, provavelmente, ocorre dado a melhor audibilidade para sons de fala e melhores condições de discriminação, determinando o SII como um preditor significativo em relação ao vocabulário receptivo^(5)^. Assim como foi encontrado nos resultados desta pesquisa, no qual o teste de vocabulário receptivo teve relação linear com o SII com AASI. Em outras palavras, ao garantir audibilidade com o uso dos dispositivos, se espera resultados melhores nos testes de linguagem, como foi apontado em pesquisas^(9)^ que sujeitos com deficiência auditiva e com boa audibilidade está diretamente relacionado com o bom desempenho de vocabulário e leitura.

A partir de outra perspectiva, o teste de vocabulário receptivo não teve correlação significativa com o SII sem AASI e no gráfico de dispersão não são observadas tendências na nuvem de pontos^(18)^. Portanto, o SII sem AASI pode ter valor preditivo, mas não pode ser considerado isoladamente como diagnóstico informativo na identificação de crianças em risco de resultados abaixo da média^(4)^.

Como indicado em outras pesquisas^(4,8-10)^, a quantidade de horas de uso do AASI isoladamente não está diretamente relacionada a habilidades de linguagem. Na presente pesquisa, ficou evidente que foi necessário um índice que associasse audibilidade – com e sem AASI – com as horas de uso para que a relação fosse significativa, isto é, a dosagem auditiva.

Para analisar a experiência auditiva das crianças considerando o quanto ela escuta quando está sem e com AASI e qual é o tempo dessa exposição, observa-se que os resultados do teste de vocabulário receptivo tendem a aumentar com o aumento da dosagem da auditiva, indicando que, quanto maior o valor da dosagem, melhores serão os resultados de linguagem receptiva. Resultados semelhantes foram apresentados em outros estudos^(3,4)^, cujos resultados são que crianças com maior dosagem auditiva apresentam resultados melhores de linguagem e melhor vocabulário receptivo do que crianças com menor dosagem auditiva.

Pode-se assim sugerir que a associação entre a dosagem auditiva e os resultados de linguagem pode ser impulsionada mais pela variabilidade no SII sem e com AASI, uma vez que a criança também está exposta aos sons de fala quando não está utilizando seus dispositivos, do que uma associação com as horas de uso do AASI^(4)^.

Assim como é demonstrado no estudo dos mesmos autores, a relação entre linguagem e dosagem auditiva é positiva em menos de 10 horas de dosagem, mas não aumenta substancialmente acima de 10 horas de dosagem e crianças com dosagem auditiva inferior a 5,3 a 6,7 corriam risco de apresentar resultados de linguagem atrasados. Embora não tenham sido usados múltiplos instrumentos na aferição de habilidades de linguagem, encontramos aqui resultados semelhantes em relação ao vocabulário receptivo medido pelo PPVT.

Garantindo a audibilidade, é possível habilitar/reabilitar as habilidades auditivas e, como consequência, possibilitando o desenvolvimento de linguagem. A terapia fonoaudiológica é de extrema importância para crianças com deficiência auditiva, uma vez que não basta apenas ter audibilidade, é necessário também um trabalho para que o reconhecimento de palavras possa refletir o desempenho compatível com a capacidade auditiva prevista pelo SII e para isso é necessário acompanhar a experiência auditiva de cada criança^(17)^.

Uma vez que crianças com melhor SII sem AASI podem acessar a fala sem o dispositivo, limitando a utilidade do uso de AASI em tempo integral em relação a crianças que têm SII menores sem AASI e que a constância do uso dos dispositivos é um fator complexo que não é diretamente indicativo da adesão efetiva^(8,10,19)^. A experiência auditiva ou dosagem auditiva podem ser utilizadas para melhorar a orientação aos pais sobre o quanto aquela criança está escutando em todas as situações, do momento que está acordada ao dormir, gerando alertas para a necessidade de possíveis mudanças no processo de reabilitação.

A coleta para essa pesquisa foi realizada juntamente com outro estudo^(10)^ e foi transversal em determinado momento do desenvolvimento de linguagem da criança, representando uma das limitações na análise. Estudos longitudinais poderiam aferir qual o efeito que horas de uso dos AASI relacionada a audibilidade tem no desenvolvimento da linguagem, possibilitando a identificação de diferenças individuais no uso dos dispositivos e variáveis intervenientes, como alterações de orelha média recorrentes ou dispositivos quebrados.

A maioria dos sujeitos da pesquisa (55,2%) apresentaram resultados dentro do escore no teste PPVT. Dentre os sujeitos que apresentaram resultado abaixo de -2 dp no teste de vocabulário receptivo padronizado (31,1%) estão S12, que frequenta escola de libras e S19 que não faz uso dos AASI. Dentre os que apresentaram resultado entre -1 e -2 dp (13,8%) está S24, sujeito com perda mista e que frequenta a escola de libras. Levando em consideração que o teste PPVT representa o cotidiano das crianças, pode-se sugerir que os sujeitos que frequentam a escola de Libras podem não ter atingido o escore do teste, uma vez que o tempo de exposição a língua oral é reduzido, considerando que seu cotidiano é em Libras.

O fato de ambos frequentarem escola especial em Libras pode ter interferido na aquisição de vocabulário na língua portuguesa oral, considerando que ambos têm pais ouvintes, o que significa que a exposição a Libras se dá principalmente no ambiente escolar. A média das frequências também não justifica a diferença nos resultados do teste, uma vez que S24 tem perda maior que S12, porém o tipo da perda auditiva difere. S24 apresentou resultados melhores, sendo que ele tem perda auditiva mista, ou seja, possui diferença entre os limiares de via óssea e de via aérea, e, em muitos casos, o componente condutivo pode ser responsável pela melhor inteligibilidade de fala.

## CONCLUSÃO

Esta pesquisa permitiu analisar os resultados de vocabulário receptivo e percepção de fala, de acordo com a experiência auditiva de crianças com deficiência auditiva, considerando a audibilidade e consistência de uso dos AASI.

Os dados sugerem:

Índices mais elevados de SII 65dB, característicos de crianças com audibilidade para mais que 56% de sons de fala, estão relacionados com o desempenho de vocabulário receptivo compatível com a faixa etária e de percepção de fala com elevados escores de desempenho, uma vez que garantindo boa audibilidade para sons de fala, os sujeitos apresentam melhores condições de discriminação;
A quantidade de horas de uso dos AASI e o SII 65 dB sem os dispositivos, isoladamente, não estão diretamente relacionadas ao desempenho no teste de vocabulário receptivo. No entanto, quando analisadas conjuntamente, com SII 65 dB com amplificação no cálculo da dosagem auditiva, explicam a variabilidade de desempenho em crianças com perdas menores;
A experiência auditiva, envolvendo a audibilidade sem e com AASI e a consistência de uso diário do dispositivo, deve ser considerada durante todo o processo de reabilitação, pois além de indicar e explicar o acesso aos sons em todas as situações, traz também elementos para orientação aos pais.

Outras pesquisas são necessárias para verificar a relação da dosagem auditiva com outros instrumentos de análise de outras habilidades de linguagem, além do vocabulário, como também estudos longitudinais para medir qual o efeito que as horas de uso do aparelho de amplificação sonora individual relacionada a audibilidade tem no desenvolvimento da linguagem oral verbal.
